# Anchoring-and-Adjustment During Affect Inferences

**DOI:** 10.3389/fpsyg.2018.02567

**Published:** 2019-01-08

**Authors:** Michelle Yik, Kin Fai Ellick Wong, Kevin J. Zeng

**Affiliations:** ^1^Division of Social Science, Hong Kong University of Science and Technology, Kowloon, Hong Kong; ^2^Department of Management, Hong Kong University of Science and Technology, Kowloon, Hong Kong

**Keywords:** anchoring-and-adjustment, affect inferences, semantic anchors, numerical anchors, time pressure

## Abstract

People can easily infer the thoughts and feelings of others from brief descriptions of scenarios. But how do they arrive at these inferences? Three studies tested how, through anchoring-and-adjustment, people used semantic and numerical anchors (irrelevant values provided by experimenters) in inferring feelings from scenario descriptions. We showed that in a between-subject design, people’s inference was biased toward anchoring information (Studies 1 and 2). People made fewer adjustments (anchoring increased) under time pressure in the high-anchor condition but not in the low-anchor condition (Study 3). When inferring affect from scenario descriptions, not only did people integrate their inference with the context, they adjusted away from the initial anchors provided by the experimenters. However, time pressure discouraged people from making adequate adjustments.

## Introduction

To navigate everyday life, people must often estimate uncertain quantities: the number of people in a long queue for a bus, the number of drinks for a party, the reasonable fee for a cruise, etc. One strategy for doing so, using what Tversky and Kahneman (1974) called the anchoring-and-adjustment heuristic, is to start with an accessible value in the context and adjust from this value to arrive at an acceptable value (quantity). To succeed in social interactions, people must gauge how others are feeling. Retirement marks the beginning of a new chapter in a person’s life. Vincent will retire after today. How would he feel? Marriage gives a couple additional family obligations. Jenny is getting married today. How would she feel? People can easily and consensually infer the protagonist’s feelings from minimal information in a scenario description (Siemer and Reisenzein, 2007; cf. Lin et al., 2010). But how exactly do people arrive at these inferences?

We propose that affect inferences are made relative to the context in situational scenarios via an anchoring-and-adjustment mechanism. Our studies might seem uncontroversial, but their implications are far-reaching. First, the process of inferring affect from verbally presented information was one of the most common platforms in emotion research (e.g., Scherer, 1999; Komeda et al., 2009; Matarazzo et al., 2014), but the mechanism behind the process has rarely been tested empirically. A better understanding of the inference process could benefit numerous fields of emotion research that deploy scenario descriptions in their studies. Second, the majority of prior social psychological research has focused on the usefulness of the anchoring-and-adjustment mechanism in inferring the sociopolitical attitudes and decision-making of others (see Tamir and Mitchell, 2013) but not in inferring affect. The present studies were designed to fill these gaps in the literature.

### Inferring Affect From Propositional Information

The present research on inferring affect by adjusting information derived from textually presented scenarios is important in advancing the psychology of emotion. Previous research has mostly focused on “non-propositional” (non-textual) cues such as facial expressions, behavioral patterns, tone of voice, and postures. In this paper, we focus on “propositional” (textual) cues. We argue that emotions communicated in propositional cues carry theoretical significance just as non-propositional cues do (Anderson, 1983; Kintsch, 1988). After all, we communicate much of our emotions on a daily basis through textually presented situational information, such as books, magazines, newspapers, and social media. It is also high time that we move beyond the facial expressions convention and explore emotions in other modalities. Our research extends prior work to an understudied modality that provides a promising testing ground for hypotheses pertinent to emotions. Would the inference of affect in scenario descriptions be influenced by the contextual information as it would be in facial expressions?

For the past century, scientists have been fascinated by the idea that “emotions are written on the face as particular arrangements of facial actions and that perceivers can read these actions as easily and effortlessly as they read words on a page” (Barrett et al., 2011, p. 286). As Matsumoto (1990) concluded, “the universality of facial expressions of emotion is no longer debated in psychology” (p. 195). Nonetheless, skeptics, unconvinced by the universality thesis, argued that emotion is, in part, read into people’s faces (Klineberg, 1938), implying that different emotions could be inferred from the same face depending on the context. Reviewing cross-cultural studies on emotion recognition among literate and illiterate perceivers, Russell (1994) and Nelson and Russell (2013) concluded that the associations between facial expressions and emotion labels varied across contexts including cultural background, response format, research design, and the preceding photographs shown to the perceivers (cf. Ekman, 1994). More recently, Barrett et al. (2011) showed that when judging emotion from a face, perceivers use any context available, including social and experimental contexts, and any emotion words available in a person’s mindset. Aviezer et al. (2011) provided convincing evidence that the face-context integration was an automatic, effortless process (cf. Masuda et al., 2008). Here, we offer new evidence by testing the effect of contextual information on affect inferences in scenario descriptions.

### The Inference Process

When the consideration of irrelevant numeric values (viz. anchors) has an influence on subsequent estimates of unknown quantities, we call this phenomenon “anchoring-and-adjustment.” The notion of anchoring-and-adjustment posits that people generate an initial estimate based on anchor values (Tversky and Kahneman, 1974). Those who consider their initial estimates “good enough” will not make any adjustments. Those who consider their initial estimates “not good enough” will adjust away from the anchor, although the adjustment is often insufficient, leading to biases toward the arbitrarily chosen anchor (Quattrone, 1982). This anchoring effect remains robust even when the anchor is completely irrelevant for the judgment task at hand. For instance, Tversky and Kahneman (1974) showed in a study that the numbers generated from the wheel of fortune were strongly correlated with participants’ estimates of the proportion of African countries in the United Nations. We tested the anchoring effect by manipulating the relevance of information to affect judgments in Studies 1 and 2.

Anchoring-and-adjustment appears to be a general mechanism behind a wide variety of judgments and decision-making in everyday life. This mechanism in particular has been instrumental in explaining the processes behind preference reversals (Schkade and Johnson, 1989), trait inference (Kruger, 1999), language comprehension (Keysar and Barr, 2002), attitude inference (Tamir and Mitchell, 2013), and various egocentric biases (Gilovich et al., 2000). Our research sought to test whether this mechanism could explain the process behind integrating affect inference with contextual information.

An important notion in the anchoring-and-adjustment mechanism is that the motivation for adjustments matters for the final judgment of affect, and that adjustment is a serial process. People start with an anchor and then adjust their inference away from that anchor with cognitive effort (Epley et al., 2004). To demonstrate the serial nature of adjustment, researchers manipulated people’s motivation (e.g., through cash incentive) to keep making adjustments. People who are motivated to give correct answers would be more willing to adjust their judgments from initial anchors than people who are unmotivated (cf. Chapman and Johnson, 2002). Thus, motivated (unmotivated) people’s final estimates would be farther away from (closer to) the anchor values. Furthermore, time also comes into play. People who are under time pressure are more likely to stop adjusting earlier than people who have sufficient time to make adjustments. We tested the serial adjustment process by manipulating the time pressure factor in Study 3.

### The Current Research

The goal of the current research was to advance our understanding of how people infer affect from propositional cues. We achieved this goal by synthesizing and extending research on two fronts: (a) elucidating the role of anchoring in affect inference in scenario descriptions, and (b) demonstrating serial adjustment as a cognitive mechanism in integrating contextual information with affect judgment.

## Study 1

The goal of Study 1 was to demonstrate the anchoring effect in people’s judgment of affect. We asked participants to read textual information and then, based on that information, to judge the protagonist’s affect. The participants were randomly assigned to either a control or an experimental condition. In both conditions, they were asked to read a piece of contextual information and then a scenario, after which they inferred the protagonist’s affect. In the control condition, we obtained the participants’ “initial judgment” by instructing them to simply judge the protagonist’s feelings in the scenario. Our intention was to get the judgments based on the contextual information. In the experimental condition, we obtained the participants’ adjustment from the initial judgment by instructing them to judge the protagonist’s affect *as if they had not read* the contextual information. In other words, the participants were encouraged to adjust their initial judgment formed by the contextual information. This condition served as a tool to demonstrate the anchoring effect, defined as the *difference in affect judgments between the control and experimental conditions* (Epley et al., 2004).

### Methods

#### Participants

The participants consisted of 183 undergraduates (112 female) studying at a university in Hong Kong.

#### Procedure

Upon arrival, each participant was assigned a computer and instructed to complete an online survey on social issues. In each session, participants were randomly assigned to either the control or experimental condition. All participants were instructed to respond to a list of 20 scenarios, 10 each for the positive and negative anchors. Each scenario was designed to appear ambiguous and thus could be interpreted as either pleasant or unpleasant. For instance, one scenario described a couple dining in a restaurant: “James and his wife were dining quietly in a restaurant.”

Preceding this scenario were two versions of the contextual information. In the positive-anchor condition, the participants were informed that “James and his wife regard *remaining silent* as an illustration of intimacy.” In the negative-anchor condition, the participants were informed that “James and his wife regard *having dialogs* as an illustration of intimacy.” After reading each scenario (and contextual information), participants were asked to rate the protagonist’s feelings.

Each participant was administered 10 scenarios preceded by the positive contextual information and another 10 by the negative contextual information. To avoid confounding the valence of the contextual information with specific scenarios, we created two counterbalanced sets of scenario descriptions (i.e., 10 scenarios in Set A and another 10 in Set B) to allow two different versions of the contextual information to be used for each of the 20 scenarios. Specifically, half of the participants were assigned to read Set A with positive contextual information and Set B with negative contextual information, whereas the remaining participants were assigned to read Set A with negative contextual information and Set B with positive contextual information. This counterbalancing procedure was to ensure that each scenario had an equal chance of being paired with the positive anchor and the negative anchor. A computer program randomly determined the order of the 20 scenarios for each participant.

#### Dependent Measure

After reading each scenario, participants rated the protagonist’s affect using four adjectives denoting pleasant feelings (*content*, *satisfied*, *at ease*, *carefree*, and *uninhibited*), culled from Yik’s (2009) measure. Ratings were made on a five-point Likert scale ranging from 1 (*not at all*) to 5 (*extremely*). For each scenario, we computed a Pleasure Index (PI) by taking the mean of the four items. For each participant, we computed a PI-positive score by averaging the PI scores across the 10 positive-anchor scenarios and a PI-negative score by averaging across the 10 negative-anchor scenarios. These PI scores were then used in subsequent analyses.

### Results

**FIGURE 1:**
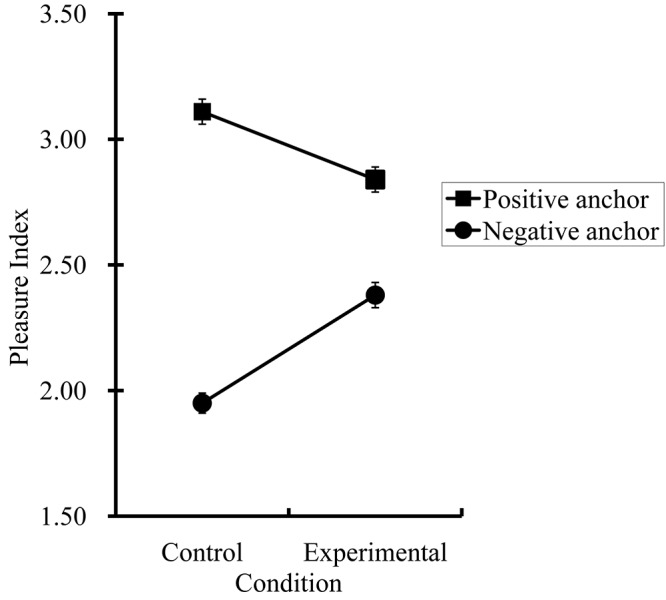
Results from Study 1 (*N* = 183): Anchoring in affect judgments. Error bars represent standard errors.

The results of Study 1 are graphed in Figure 1 and the descriptive statistics are summarized in Table 1. Two observations were made: (a) a significant anchoring effect was found in the control condition, and (b) the anchoring effect in the experimental condition was smaller than that in the control condition. These observations were then examined with a 2 (condition: control vs. experimental) × 2 (anchor: positive vs. negative) mixed-model analysis of variance (ANOVA) with repeated measures on the last factor. This ANOVA revealed a significant anchoring effect on the mean PI scores, *F*(1,181) = 370.24, *p* < 0.01, $\eta_{p}^{2}$ = 0.67 (95% Confidence Interval [95 CI]: 0.60–0.73), qualified by a two-way interaction, *F*(1,181)_anchor__×__condition_ = 67.87, *p* < 0.01, $\eta_{p}^{2}$ = 0.27 (95 CI: 0.17–0.37).

**Table 1:** Mean PI scores by anchor and condition in studies 1 and 2.

Condition	PI-positive		PI-neutral		PI-negative	
	*M*	*SD*	*M*	*SD*	*M*	*SD*
**Study 1**						
Control (*n* = 93)	3.11	0.49	–	–	1.95	0.37
Experimental (*n* = 90)	2.84	0.48	–	–	2.38	0.50
**Study 2**						
Control (*n* = 76)	3.13	0.49	2.63	0.52	1.99	0.41
Experimental (*n* = 76)	2.79	0.50	2.59	0.47	2.38	0.51


*PI, pleasure index. PI scores range from 1.00 to 5.00. PI-positive, PI for positive anchors; PI-neutral, PI for neutral anchors; PI-negative, PI for negative anchors. Neutral anchors were not used in Study 1.*

Next we performed two planned comparisons (i.e., lowering the alpha value from 0.05 to 0.03 by Bonferroni’s adjustment). The comparisons revealed that the PI-positive score was higher than the PI-negative score in the control condition, *F*(1,181) = 383.86, *p* < 0.01, $\eta_{p}^{2}$ = 0.68 (95 CI: 0.60–0.73), and that the difference remained significant in the experimental condition, *F*(1,181) = 59.56, *p* < 0.01, $\eta_{p}^{2}$ = 0.25 (95 CI: 0.15–0.35).

In Study 1, the difference in the anchoring effect between the control and experimental conditions demonstrated that the participants formed an initial judgment about the protagonist’s affect based on the contextual information (the control condition) and that when instructed to ignore the contextual information (the experimental condition), participants moved their judgment away from the initial anchor. One drawback in Study 1 was that the anchoring manipulation did not have a neutral anchor condition (baseline). We were unsure if the observed anchoring pattern was driven by the positive anchors, the negative anchors, or both. More specifically, these results offered three possible interpretations. First, it is possible that the difference was due to people’s judgment being assimilated toward positive anchors but not negative anchors (i.e., anchoring driven by positive anchors only). Second, it is possible that the difference was due to people’s judgment being assimilated toward negative anchors but not positive anchors (i.e., anchoring driven by negative anchors only). Third, it is possible that both positive and negative anchors exerted the anchoring effects. Theoretically, anchoring effects should be driven by both positive and negative anchors. We inserted a neutral baseline condition in Study 2 to test these possibilities.

## Study 2

### Methods

#### Participants

The participants consisted of 152 undergraduates (96 female) studying at a university in Hong Kong.

#### Procedure

The procedures and materials were identical to those of Study 1 except the following. First, we inserted a neutral anchor condition for each scenario in which the contextual information did not imply any valence. Second, we added one more scenario for a total of 21 scenarios. Third, we prepared three (not two) counterbalanced sets of scenario descriptions. As such, we had in each set seven scenarios corresponding to the positive-anchor condition, seven to the negative-anchor condition, and seven to the neutral-anchor condition. We estimated the PI-positive, PI-negative scores, and PI-neutral scores.

### Results

**FIGURE 2:**
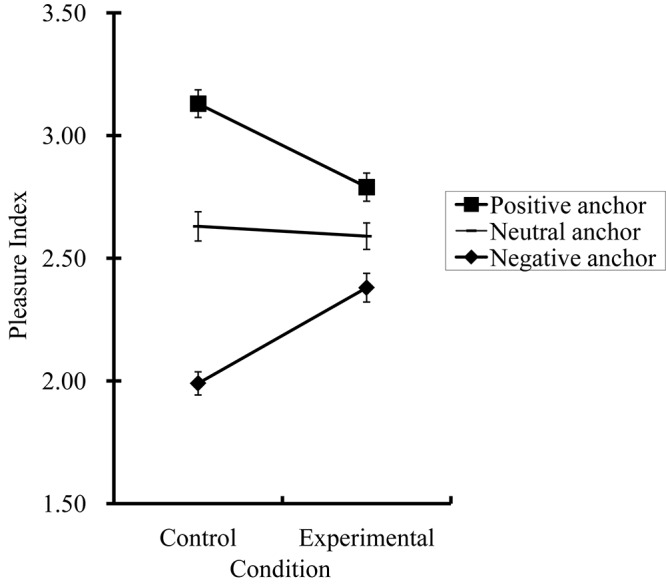
Results from Study 2 (*N* = 152): Anchoring in affect judgments as a function of contextual information. Error bars represent standard errors.

The results of Study 2 are graphed in Figure 2 and the descriptive statistics are summarized in Table 1. The patterns of results are very similar to those found in Study 1: (a) the anchoring effect was found in the control condition, and (b) the anchoring effect in the experimental condition was smaller than that in the control condition. These observations were then examined with a 2 (condition: control vs. experimental) × 3 (anchor: positive vs. negative vs. neutral) mixed-model ANOVA with repeated measures on the last factor. This ANOVA revealed a significant anchoring effect on the PI scores, *F*(2,300) = 169.86, *p* < 0.01, $\eta_{p}^{2}$ = 0.53 (95 CI: 0.46–0.59), qualified by the predicted interaction, *F*(2,300)_condition__×__anchor_ = 37.93, *p* < 0.01, $\eta_{p}^{2}$ = 0.20 (95 CI: 0.12–0.28).

To further understand the interaction effect, we performed two comparisons with alpha = 0.01 after applying Bonferroni’s adjustment. In the control condition, planned comparisons revealed that the PI-positive score (*M* = 3.13, *SD* = 0.49) was higher than the PI-neutral score (*M* = 2.63, *SD* = 0.52), *F*(1,150) = 79.54, *p* < 0.01, $\eta_{p}^{2}$ = 0.35 (95 CI: 0.22–0.43), whereas the PI-negative score (*M* = 1.99, *SD* = 0.41) was lower than the PI-neutral score, *F*(1,150) = 117.86, *p* < 0.01, $\eta_{p}^{2}$ = 0.44 (95 CI: 0.32–0.53). The effect of the contextual information (i.e., the anchor) was as predicted. When given positive, negative, and neutral anchors, the participants interpreted the protagonist as experiencing pleasant, unpleasant, and neither pleasant nor unpleasant feelings, respectively.

In the experimental condition, the PI-positive score (*M* = 2.79, *SD* = 0.50) was higher than the PI-neutral score (*M* = 2.59, *SD* = 0.47), *F*(1,150) = 12.78, *p* < 0.01, $\eta_{p}^{2}$ = 0.08 (95 CI: 0.02–0.17), whereas the PI-negative score (*M* = 2.38, *SD* = 0.51) was lower than the PI-neutral score, *F*(1,150) = 12.64, *p* < 0.01, $\eta_{p}^{2}$ = 0.08 (95 CI: 0.02–0.17). These results indicate that even when participants were instructed to ignore the anchors, those who had received positive anchors still interpreted the protagonists as being happier than did those who had received negative anchors.

## Study 3

As the name implies, the anchoring-and-adjustment mechanism involves first the anchoring component, followed by the adjustment component. Nonetheless, the literature has primarily taken an interest in the anchoring component, while sidelining the adjustment component (cf. Epley and Gilovich, 2006; Tamir and Mitchell, 2013). Epley and Gilovich suggested that adjustment might not be as robust as anchoring, because adjustment appears to be relatively weak especially for the anchors provided by the experimenters, which were what we used in Study 3.

The significant anchoring effects reported in Studies 1 and 2, although focusing on a non-traditional domain (viz. affect inferences) in the pertinent literature, are not too surprising. These studies did not directly test the adjustment component of the anchoring-and-adjustment mechanism. Was the adjustment component involved in the inferences? In Study 3, we tested the adjustment process by using time pressure as an adjustment manipulation, and numerical anchors. Adjustment takes time because it involves evaluating different hypotheses on how much the initial anchor deviates from the true value. We predicted that responses made under time pressure would be closer to the initial anchor than those made with no time limit.

Study 3 addressed two more limitations of Studies 1 and 2. First, contextual information served as the anchor in the first two studies, but this information was semantic in nature, whereas in the anchoring literature, anchors of a numeric nature are more often used. We used semantic information and simply assumed that people would generate their own numerical anchors based on this information (Epley and Gilovich, 2006). To cross-validate the anchoring effects found in Studies 1 and 2, we used experimenter-provided numerical anchors in Study 3. Second, the participants in the prior studies might regard the contextual information as cues for the subsequent judgment, despite our request to ignore this information in the experimental condition. The essence of anchoring-and-adjustment is that irrelevant, arbitrary information in the environment serves as the starting point in a judgment process (Tversky and Kahneman, 1974; Wilson et al., 1996; Wong and Kwong, 2000). In Study 3, we sought stronger evidence for the anchoring effect by using numerical anchors that were irrelevant to the scenario descriptions.

### Methods

#### Participants

Participants in Study 3 consisted of 205 undergraduates (99 female) studying at a university in Hong Kong.

#### Procedure

Upon arrival, each participant was assigned a computer with an earphone (beep sounds were played) and instructed to infer the feelings of the protagonist, on a scale of 1 (*not at all*) to 9 (*extremely*), for the same 21 scenarios used in Study 2.

On each web page, the participants first read a scenario about a fictitious protagonist and were asked to rate how the protagonist felt. Prior to making their own judgments, participants were presented with “sample answers” to demonstrate how to complete the ratings. The sample answers contained numerical ratings to the four pleasure terms used in Study 1. The numerical ratings served as an anchor to influence people’s judgment of the protagonist’s affect. One hundred and one participants were randomly assigned to the “low-anchor” condition in which the sample ratings were all 1 (the low end of the scale); the remaining 104 participants were assigned to the “high-anchor” condition in which the sample ratings were all 9 (the high end of the scale).

In each anchor condition, the participants were further assigned to either the time-pressure condition (the task to be completed within 25 s) or the control condition (no time limit). Participants were given 5 s to read the scenario, the questions, and the sample answers. Subsequently, a message on the screen reminded them to give their four ratings immediately after hearing a 1-s beep sound. They could then continue to the following page by pressing the “next page” button.

In the time-pressure condition, a black bar appeared for 25 s below the text message prompting the participants to complete all four ratings. At the end of the 25 s, a long beep sound would be played and the screen would automatically jump to the following page even if the participants had not finished all the ratings. Participants who did not respond within 25 s were excluded from analyses. In the control condition, participants could take as long as they wished to complete their ratings. The system recorded the time that the participants spent on each web page, defined as the number of seconds from when the pleasure items were first displayed to when the “next page” button was clicked.

### Results

The 13 cases in which the participants failed to complete all the ratings within the time limit in the time-pressure condition were dropped. Subsequent analyses were based on the remaining 192 cases. The time-pressure manipulation was successful. It took participants less time to complete ratings in the time-pressure condition (*M* = 14.01 s, *SD* = 3.56 s) than in the control condition (*M* = 18.15 s, *SD* = 5.62 s), *F*(1,190) = 36.72, *p* < 0.01, $\eta_{p}^{2}$ = 0.17 (95 CI: 0.08–0.25). The response time, however, did not differ between the low-anchor and high-anchor conditions and thus was not related to which anchor the participants received.

For each scenario, we computed a PI by taking the mean of the four pleasure ratings. For each participant, we computed a mean PI score by averaging the PI scores across the 21 scenarios. This mean score was then used in subsequent analyses.

**FIGURE 3:**
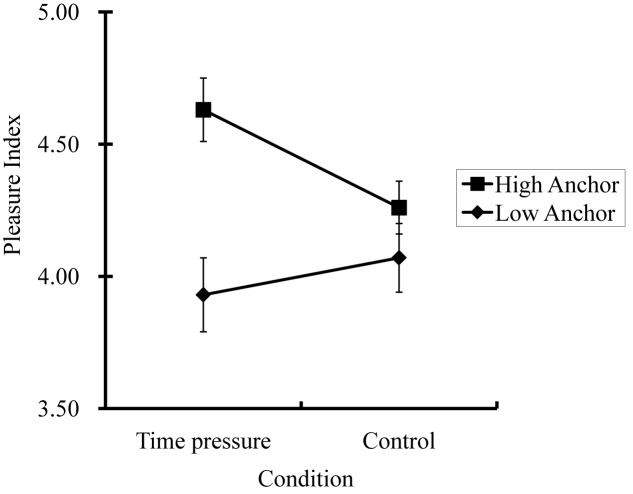
Results from Study 3 (*N* = 192): Adjustments in affect judgments away from sample answers. Error bars represent standard errors.

The results of Study 3 are graphed in Figure 3 with PI as the dependent variable. Descriptive statistics are summarized in Table 2. We observed a significant anchoring effect in the time-pressure condition but not in the control condition, presumably because time pressure prevented people from adjusting their estimates away from the anchor values. This observation was supported by a 2 (condition: time pressure vs. control) × 2 (anchor: high vs. low) two-way factorial ANOVA, which yielded a significant anchoring effect, *F*(1,188) = 13.11, *p* < 0.01, $\eta_{p}^{2}$ = 0.07 (95 CI: 0.01–0.14), qualified by a two-way interaction, *F*(1,188)_condition__×__anchor_ = 4.36, *p* = 0.04, $\eta_{p}^{2}$ = 0.02 (95 CI: 0.00–0.08).

**Table 2:** Mean PI scores by anchor and condition in study 3.

Condition	Low anchor			High anchor		
	*n*	*M*	*SD*	*n*	*M*	*SD*
Time pressure	48	3.93	0.94	46	4.63	0.82
Control	49	4.07	0.92	49	4.26	0.72


*PI, pleasure index. PI scores range from 1.00 to 9.00.*

We made four mean comparisons with alpha = 0.01 after applying Bonferroni’s adjustment. We found that the difference between low-anchor and high-anchor ratings was significant in the time-pressure condition, *F*(1,188) = 15.96, *p* < 0.01, $\eta_{p}^{2}$ = 0.08 (95 CI: 0.02–0.16), but not in the control condition, *F*(1,188) = 1.20, *p* = 0.27. These findings are consistent with our hypothesis that people make serial adjustments away from experimenter-provided anchors in affect judgment. Because of the time limit, the participants in the time-pressure condition made fewer adjustments away from the provided anchors, thereby exhibiting more anchoring effect, than did the participants in the control condition.

To test the adjustment in each anchor condition, we next conducted planned comparisons between the mean values of PI in the time-pressure condition and the control condition. The difference between the two conditions was marginally significant in the high-anchor condition, *F*(1,188) = 4.51, *p* = 0.04, $\eta_{p}^{2}$ = 0.02 (95 CI: 01.00–0.08), but not in the low-anchor condition, *F*(1,188) = 0.68, *p* = 0.41. The pattern of results shows that the participants made significant serial adjustments in the high-anchor condition from the time-pressure to the control conditions. A similar effect, however, was not found in the low-anchor condition.

## General Discussion

The social psychology literature has long documented that judgments are affected by contextual information (Helson, 1964; Parducci, 1965). Our research was first and foremost designed to study the cognitive mechanism behind the process of affect inferences using the anchoring-and-adjustment mechanism. Not only did we examine the effect of contextual information on the judgments, we looked for ways to influence these judgments. Our data support that affect judgments initially are made using experimenter-provided anchors and subsequently adjusted.

The notion of anchoring-and-adjustment predicts that motivation encourages people to adjust their judgments – whether closer to or further away from a given anchor. A considerable amount of research has been conducted to test how anchors influence judgments, and alternative theories for different attributes of anchors have been developed. In many of these studies, motivation was found to influence adjustments if the anchors were generated by the participants themselves (e.g., “What year did Britain take over Hong Kong?”) but not if they were provided by the experimenter (e.g., “Did Britain take over Hong Kong after 1880?”) (see Strack and Mussweiler, 1997; Chapman and Johnson, 2002; Epley and Gilovich, 2006). In our studies, all anchors were provided by the experimenters and were found to influence affect inferences. Future studies should be conducted to cross-validate the present results in other social judgments.

In the current research, we used (non-traditional) semantic and (traditional) numerical anchors. In Studies 1 and 2, we gave the participants no numerical information. The initial values therefore were inferred from the contextual information. In Study 3, following the anchoring literature, we gave specific random numerical values. Despite the different attributes of the anchors, all three studies produced anchoring effects through the experimenter-provided anchors (cf. Epley and Gilovich, 2006). We computed the effect sizes of the anchoring, which varied across conditions (*d* = 0.93 in Study 1’s experimental condition, *d* = 0.81 in Study 2’s experimental condition, *d* = 0.80 in Study 3’s time-pressure condition, and *d* = 0.23 in Study 3’s control condition). Because we assumed that a numeric-to-numeric connection would be stronger than a semantic-to-numeric connection, we initially expected that semantically induced anchoring (Studies 1 and 2) would be weaker than numerically induced anchoring (Study 3). However, our findings proved us wrong. Because the studies were not designed to examine the effect of semantic versus numeric anchors, the anchors deployed varied in many other ways. Future research should address this issue by directly comparing anchoring effects with better controlled designs.

We observed in Study 3 an interesting asymmetry between high anchors and low anchors, with high anchors being more vulnerable to the effect of time pressure. A similar asymmetrical anchoring effect was first noted by Jacowitz and Kahneman (1995), who found that high anchors induced stronger adjustment effects than did low anchors. Subsequent anchoring studies (Wilson et al., 1996; Wong and Kwong, 2000), therefore, often chose only high anchors so as to maximize the size of the anchoring effect. However, researchers have yet to understand how and why the high–low anchoring asymmetry exists. The asymmetry effect observed in Study 3 reinforces our proposition that affect inference involves an anchoring-and-adjustment mechanism but also offers new insights into the locus of the asymmetry. Specifically, the high–low anchoring asymmetry exists in two loci: at an earlier processing stage of activating a number adjacent to the anchor value and selecting that number as the starting candidate (Wong and Kwong, 2000); and at a later processing stage of adjusting from the starting candidate (Epley et al., 2004). Because time pressure should influence the adjustment but not the activation, the stronger effect of time pressure on the high (vs. low) anchor condition in Study 3 implies that the locus likely lies in the adjustment but not the anchoring process. Future research should investigate the factors driving the adjustment process.

Finally, we excluded 13 participants in Study 3 due to their slow responses. This exclusion happened in the time-pressure condition but not in the control condition. Because this exclusion was not random, it may create a selection bias, rendering the manipulation as not completely random. With only a small proportion of participants excluded in Study 3, we expect that this exclusion is not a serious problem for our general findings and interpretation. Nonetheless, future research should replicate Study 3 with less time pressure, allowing for fewer exclusion cases.

## Ethics Statement

The studies reported in this paper were carried out in accordance with the APA ethical standards. The protocol was approved by the HKUST Human Participants Research Panel. All subjects gave written informed consent in accordance with the Declaration of Helsinki.

## Author Contributions

MY and KW focused on designing the experiments and shaping up the entire manuscript for publication. KZ worked on the experiment materials, data collection and analyses, and preparation of the results section.

## Conflict of Interest Statement

The authors declare that the research was conducted in the absence of any commercial or financial relationships that could be construed as a potential conflict of interest.
